# Comprehensive and simultaneous coverage of lipid and polar metabolites for endogenous cellular metabolomics using HILIC-TOF-MS

**DOI:** 10.1007/s00216-014-7797-5

**Published:** 2014-04-10

**Authors:** Fan Fei, Dawn M. E. Bowdish, Brian E. McCarry

**Affiliations:** 1 Department of Chemistry and Chemical Biology, McMaster University, Hamilton, L8S4M1 Canada; 2 Department of Pathology and Molecular Medicine, Michael G. DeGroote Institute for Infectious Disease Research, McMaster University, Hamilton, L8N3Z5 Canada

**Keywords:** Comprehensive metabolomics, Polar metabolites, Lipids, HILIC-TOF-MS, Bacteria, Macrophage

## Abstract

The comprehensive metabolomic analyses using eukaryotic and prokaryotic cells are an effective way to identify biomarkers or biochemical pathways which can then be used to characterize disease states, differences between cell lines or inducers of cellular stress responses. One of the most commonly used extraction methods for comprehensive metabolomics is the Bligh and Dyer method (BD) which separates the metabolome into polar and nonpolar fractions. These fractions are then typically analysed separately using hydrophilic interaction liquid chromatography (HILIC) and reversed-phase (RP) liquid chromatography (LC), respectively. However, this method has low sample throughput and can also be biased to either polar or nonpolar metabolites. Here, we introduce a MeOH/EtOH/H_2_O extraction paired with HILIC-time-of-flight (TOF)-mass spectrometry (MS) for comprehensive and simultaneous detection of both polar and nonpolar metabolites that is compatible for a wide array of cellular species cultured in different growth media. This method has been shown to be capable of separating polar metabolites by a HILIC mechanism and classes of lipids by an adsorption-like mechanism. Furthermore, this method is scalable and offers a substantial increase in sample throughput compared to BD with comparable extraction efficiency. This method was able to cover 92.2 % of the detectable metabolome of Gram-negative bacterium *Sinorhizobium meliloti*, as compared to 91.6 % of the metabolome by a combination of BD polar (59.4 %) and BD nonpolar (53.9 %) fractions. This single-extraction HILIC approach was successfully used to characterize the endometabolism of Gram-negative and Gram-positive bacteria as well as mammalian macrophages.

**Electronic supplementary material:**

The online version of this article (doi:10.1007/s00216-014-7797-5) contains supplementary material, which is available to authorized users.

**Figure:**
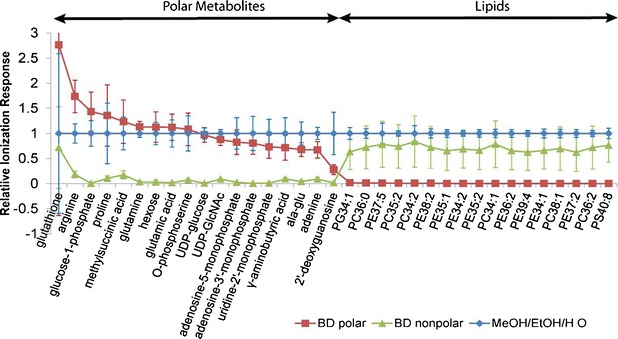
The extraction and ionization efficiency of MeOH/EtOH/H2O HILIC approach encompasses both the polar and nonpolar fractions from Bligh and Dyer extraction

## Introduction

Cellular metabolomics is an important part of systems biology as it reflects the phenotype of cells and monitors cellular activities in a perturbed system [1, 2]. The metabolomic profile of any organism represents a snapshot of its physiological state and reflects the overall contributions from genomic, transcriptomic, proteomic, and other environmental factors [3]. In vitro studies with cell cultures are convenient, fast, cost-effective and more controllable compared to animal studies using sera, tissues or body fluids, yet are still able to provide insight into biological functions [4]. Therefore, cellular metabolomics is capable of providing an understanding of the global biochemical behaviour of a biological system.

Comprehensive cellular metabolomics facilitates observation-driven, hypothesis-generating experiments by examining the entire detectable metabolome of a cellular system in order to discover new biochemical phenomena [5]. Comprehensive metabolomics involves the analyses of diverse chemical classes including but not limited to sugars, nucleotides and organic acids with varying polarity, solubility and volatility. The endogenous cellular concentration of many metabolites can span over 12 orders of magnitude (from mM to fM) [6, 7]. Expanding the diverse detectable metabolome over a broad range of concentration, physical and chemical properties is challenging yet crucial since multiple pathways can often be influenced by a single external variable. In particular, central carbon metabolism and lipid metabolism are both involved in the inflammatory response of murine macrophages undergoing lipopolysaccharide stimulation [8–11]. The quality of the results often relies on the extent of metabolome coverage in order to unveil the biochemical changes entirely.

Liquid chromatography combined with mass spectrometry equipped with an electrospray ionization source (LC-ESI-MS) is the predominant analytical methodology used for comprehensive metabolomics [12–14]. The dynamic range of time-of-flight (TOF) MS is often greater than 3 orders of magnitude [15] and affords untargeted detection of relatively abundant metabolites with little sample pretreatment. The detected metabolite features, including ion source fragments, adducts and isotopic ions, are defined by a unique combination of *m*/*z* and retention time value. The relative abundances of the collective list of metabolite features are used to evaluate and compare different treatments [16]. Many comprehensive metabolomics reports to date are focused on either the polar or lipid fractions of the metabolome. The few studies that reflect true comprehensive metabolome have analysed the polar fraction using hydrophilic interaction liquid chromatography (HILIC) and the lipid nonpolar fraction using reversed-phase (RP) LC with C18 or C8 columns [17–20]. The polar and lipid fractions are conventionally extracted from a biological sample using the Bligh and Dyer (BD) method [21]. There is also a single extraction-dual separation LC-MS for the analysis of a single extract containing both polar and nonpolar metabolites separately on HILIC and RPLC [16]. Both methods are able to ensure global coverage of the metabolome; however, these methodologies are time consuming in sample preparation and analysis.

Here, we present a reproducible, high-throughput, single-extraction HILIC approach for comprehensive cellular metabolomic analysis. This approach is able to simultaneously analyse a broad range of polar and nonpolar metabolites, which is ideal for a large-scale hypothesis-generating study with twofold faster data acquisition and analysis time when compared to the conventional methods. The metabolome coverage of the scalable extraction was compared to both the polar and the nonpolar fractions of BD method. This approach has been successfully applied to three different cell types: the Gram-positive bacterium *Streptococcus intermedius*, the Gram-negative bacterium *Sinorhizobium meliloti* and mammalian macrophages. The overall metabolome coverage observed with this single-extraction HILIC approach is equivalent to the BD method with separate analyses of polar and nonpolar fractions.

## Experimental

### Chemicals

HPLC-grade methanol (MeOH), ethanol (EtOH), acetonitrile (ACN), chloroform (CHCl_3_) and water (H_2_O) were purchased from Caledon laboratories (Georgetown, ON, Canada). Ammonium acetate and formic acid were purchased from Fisher Scientific Company (Ottawa, ON, Canada). The 2.0-mm steel chrome ball bearings were purchased from Bearing & Oil Seals Specialists Inc. (Hamilton, ON, Canada). The isotopically labelled standards for recovery determination (RS) and for peak intensity normalization (IS) were purchased from Cambridge Isotope Laboratories (Andover, MA, USA). Lipid standards were purchased from Avanti® Polar Lipids, Inc. (Alabaster, AL, USA), and other chemical standards for LC-MS were purchased from Sigma Aldrich (St. Louis, MO, USA) and Biolog Inc. (Hayward, CA, USA). The full list of metabolite standards can be found in the Electronic Supplementary Material Table S1.

### Cell culture and collection

Detailed growth conditions for the Gram-negative bacterium, *S. meliloti*, the Gram-positive bacterium, *S. intermedius*, and murine macrophages are included in the Electronic Supplementary Material Section 1. Detailed discussion regarding quenching methods for cellular metabolism and detachment protocol for adherent cells can be found in the Electronic Supplementary Material Sections 2–5 and Fig. S1. The entire harvesting and extraction process was performed on ice or in a 4 °C cold room.

#### Suspension bacterial cell culture

Cells were cultured in either 5-mL autoclaved test tubes or in sterile 96-well plates (Corning® Costar®, NY, USA). The suspension cell cultures were centrifuged at 9,500×*g* (13,000 rpm) at 4 °C in a Beckman Coulter Allegra X-22R centrifuge for 3 min, and the supernatants were carefully aspirated with micropipette and discarded. The cell pellet was resuspended in 1 mL of cold saline solution (0.85 % NaCl) or phosphate-buffered saline (PBS). The mixture was centrifuged at 9,500×*g* for 3 min, and the wash solvent was aspirated and discarded. Extraction solvent of 100 μL (1:2:1 MeOH/CHCl_3_/H_2_O, 1:1 MeOH/EtOH or 2:2:1 MeOH/EtOH/H_2_O) containing RS was added to the washed cell pellet which was then extracted immediately.

#### Adherent macrophage cell culture

For adherent macrophage cultures in 6-well tissue culture plates (Falcon®, NY, USA), the growth medium was aspirated carefully with a micropipette. The cells were quickly washed with 1 mL of cold saline or PBS. After removing the wash solvent via aspiration, 200 μL of extract solvent (1:1 MeOH/H_2_O for BD, 1:1 MeOH/EtOH or 2:2:1 MeOH/EtOH/H_2_O) containing RS was added to each well. Cells were detached from the culture plate using a cell lifter in the presence of the extraction solvent, and the cell mixture was transferred into a 1.5-mL microtube (Diamed, Mississauga, ON, Canada). Only for the Bligh and Dyer extraction, 200 μL volume of CHCl_3_ was also added to the microtube, and the mixture was extracted immediately. The volume of the extraction solvent should be adjusted to the size of the well if other types of microtiter plates were used.

### Intracellular metabolite extraction

The extraction protocol was optimized using *S. meliloti* (2 × 10^9^ cells) and applied to *S. intermedius* (1 × 10^9^ cells) as well as murine macrophages (3 × 10^5^ cells). RS were prepared such that the final concentrations of l-methionine-d_3_, l-tryptophan-d_5_ and l-lysine-^13^C_6_-^15^ N_2_ were 27, 24 and 20 μM, respectively, in the final reconstituted cell extracts. The final extracts were blown down to dryness under a gentle stream of nitrogen gas and reconstituted in 50 μL of 60 %*v*/*v* ACN/H_2_O containing l-phenylalanine-d_8_ (25 μM), Gly-Phe (16 μM), Phe-Phe (6 μM) as well as cytidine-ribose^13^C_5_ (20 μM) as IS. To minimize variability, sample extractions, addition of IS and preparation of quality control (QC) samples were prepared in the same day using the same batch of solvents. The final endogenous cellular extracts were stored at −80 °C freezer until HILIC-ESI-TOF-MS analyses.

#### Bligh and Dyer extraction

The extraction process was based on the method of Bligh and Dyer [21] to achieve a biphasic separation by using 1:2:1 MeOH/CHCl_3_/H_2_O as the extraction solvent. The top MeOH/H_2_O fraction contained polar metabolites, and the bottom CHCl_3_ fraction contained nonpolar metabolites (i.e. lipids). The denatured proteins and other macromolecules, such as DNA and RNA, were precipitated and suspended at the interface between polar and nonpolar fractions. The cell mixture was vigorously mixed by vortex for 2 min in the presence of two 2.0-mm ball bearings. After removal of the bearings, the cell mixture was centrifuged at 9,500×*g* for 3 min. The polar fraction was collected, and the protein film and the lipid fraction were re-extracted twice with 50 μL of cold MeOH/H_2_O (1:1) with the same ball bearings. The combined polar fraction was back-extracted twice with 50 μL of cold CHCl_3_, and the nonpolar CHCl_3_ fractions were collected and combined with remaining CHCl_3_ fraction from the previous polar extraction. In total, 150 μL of polar and nonpolar fractions of bacterial extracts or 300 μL from adherent cell cultures was collected.

#### MeOH/EtOH/H_2_O extraction

This extraction protocol was based on a plasma extraction procedure adopted from Bruce et al. [22]. An extraction solvent of 2:2:1 MeOH/EtOH/H_2_O or 1:1 MeOH/EtOH was used to generate a single fraction which contained a mixture of polar and nonpolar lipid metabolites. Proteins and other macromolecules were precipitated. Prior to extraction, 10 μL of RS was added to the cell mix. The cell mixture was mixed rigorously by vortex for 2 min in the presence of two 2.0-mm ball bearings. After removal of the bearings, the mixture was centrifuged at 9,500×*g* for 3 min. The supernatant was collected, and the precipitated pellet (containing DNA, RNA and proteins) was re-extracted twice with 50 μL of the cold corresponding extraction solvent with bead beating. A total of 150 μL cell extracts was collected for suspended cell cultures, or 300 μL was collected for adherent cell culture.

### LC-MS analysis

The endogenous cellular extracts were analysed using an Agilent Technologies 1200 RR Series II LC coupled to a Bruker MicrOTOF II Mass Spectrometer. An injection of 2 μL was separated on a 50 mm × 2.1 mm Kinetex 2.6 μm HILIC column of a pore size of 100 Å (Phenomenex, CA, USA). The mobile phases were HPLC-grade acetonitrile (A) and 10 mM ammonium acetate in HPLC-grade water adjusted to pH 3 with formic acid (B) at a flow rate of 200 μL/min. The column temperature was maintained at 40 °C, and the auto-sampler storage tray was set at 4 °C. The mobile phase gradient eluted isocratically with 95 % ACN for 0.5 min followed by a gradient to 35 % ACN over 12 min. The gradient was maintained at 35 % ACN for 0.5 min and increased to 95 % ACN over 1 min. The gradient was then followed by a 10-min re-equilibration prior to the next injection. The total time for the HILIC gradient was 24 min. Positive ionization mode (ESI+) and negative ionization mode (ESI−) were performed in separate runs. Details of the optimization of the HILIC gradient method using 2^3^ full factorial design can be found in the Electronic Supplementary Material Section 6 and Fig. S2.

The parameters chosen for ESI conditions were as follows: 4.0 bar of nebulizer pressure; −500 V of endplate offset; −3,800 or 4,500 V of capillary voltage; 8.0 L/min of drying gas flow rate; and 250 °C of dry gas temperature. The data were acquired in profile mode from 50 to 1,000 *m*/*z* at a scan rate of 1.0 Hz (computed using a rolling average value of 2). The mass accuracy was adjusted by internal calibration using endogenous sodium formate clusters in both ESI+ and ESI− with Bruker’s data analysis software.

Each of the extraction methods was repeated in sextuplicate, and all of the samples were analysed in random order. A pooled QC sample was prepared by combining equal volumes of all samples and divided into individual aliquots after thorough mixing. The QC samples were stored in −80 °C freezer along with the samples. A new QC sample and a day’s worth of samples (about 20 samples) were thawed and ran each day for a multiday experiment. The QC sample was injected five times at the beginning of the analysis to condition the column and also injected after every five samples. A solvent blank (e.g. methanol) and a standard mixture containing all IS and RS were also run after every ten samples. Post-column addition with Gly-Phe was performed on all different matrices for different cell types in both ESI+ and ESI− modes for ion suppression studies.

### Data analysis

Raw data obtained from LC-ESI-mircOTOF-MS were converted to the .mzXML file format using BrukerCompassxport (http://www.bdal.com/navi/meta/home.html) after internal calibration. The .mzXML files were then processed with XCMS [23, 24] and CAMERA [25] in R Project (version 2.12.2). A tabulated metabolite feature list with aligned retention time and *m*/*z* values was exported in .csv format. For XCMS, the centWave algorithm [24] was used for peak picking with a resolution of 30 ppm and a signal-to-noise threshold (snthr) set to 10. Features that appeared in less than 80 % of the samples which underwent the same extraction method were removed (minfrac = 0.8). The isotopic ions, in-source fragments, and adducts were identified using CAMERA.

Features with apparent retention factors *k*_app_′ lower than 0.7 were removed because these were not retained and experienced great ion suppression. The isotopic ions, ions associated with IS, RS and sodium formate clusters were removed. Metabolite features detected in the biological samples were compared to those in the IS and RS solution in 60 %*v*/*v* ACN/H_2_O, and the duplicated ions that were associated with the background noise were removed. The peak areas of all metabolite features were normalized using the peak area of IS according to their retention time (see Electronic Supplementary Material Fig. S3). In-source fragments and adducts were treated as separate metabolite features. Metabolite features with peak areas under 2,000 were excluded. Features with greater than 30 % variance in QC samples were removed. Integration of non-Gaussian or coeluting peaks using XCMS may generate inconsistent results. IS and RS with greater than 10 % variance and significantly differentiated metabolite features with greater than 20 % variance were re-evaluated with manual integration using Bruker DataAnalysis 4.0.

The processed data sets were used as an input for SIMCA-P+ 11 software (Umetrics, Kinnelon, NJ). Pareto scaling was applied prior to principal component analysis (PCA) and orthogonal partial least-squares discriminative analysis (OPLS-DA). OPLS-DA was used to differentiate metabolite profiles between different extraction methods. The model validation parameter *Q* ^2^ (the fraction of variations of *X* and *Y* matrices explained by the model; the *X* matrix was the metabolite features, and the *Y* matrix was the treatment groups) values above 0.4 were indicative of a robust model, i.e. true differences between the comparing groups, and *Q* ^2^ between 0.7 and 1.0 indicated that the model was highly robust [26]. *R* ^2^ *X* (*R* ^2^ *Y*) indicated the fraction in which *X* (*Y*) matrix was explained by the model. Two-tailed, unpaired heteroscedastic Student’s *t* tests with *p* < 0.05 were computed in Microsoft Excel 2010 and used to identify metabolite features that were significantly differentially expressed in each extraction method. Metabolite features were identified by matching the *m*/*z* and retention value to the available authentic standards. Figures were created in Adobe Illustrator CS5.

## Results and discussion

### Comparison of extraction solvents

There have been extensive reviews that compare various different extraction strategies for cellular metabolic analyses [27–29]. Here, we have focused on comparing the extraction efficiency of both polar and nonpolar metabolites with biphasic solvent MeOH/H_2_O/CHCl_3_ (1:1:2) in the BD method [21] to two other extraction solvents, MeOH/EtOH (1:1) and MeOH/EtOH/H_2_O (2:2:1). BD extraction is commonly used for comprehensive metabolomics to ensure full coverage of both polar and nonpolar metabolites by running both polar and nonpolar phases separately on HILIC and RPLC. Similar to the BD method, MeOH/EtOH and MeOH/EtOH/H_2_O were able to extract both polar and nonpolar metabolites, but with all metabolites present in one single phase. Therefore, the latter two extraction strategies, when compared to BD, were able to minimize sample handling and also shorten the analysis time by a factor of two while still maintaining comprehensive coverage of both polar and nonpolar metabolites.

The scalable extraction method was tested on a 100-μL *S. meliloti* (2 × 10^9^) cell culture grown in M9 growth medium in a 96-well microtiter plate. A bead-beating technique was adopted instead of vortex mixing to ensure full cell disruption. Sonication, though allowing complete cell disruption, was not used in order to avoid overheating which may degrade thermally labile metabolites. All three extraction solvents were able to precipitate protein, DNA and RNA as well as providing good recovery for a wide range of both hydrophilic and hydrophobic metabolites.

Based on the methionine-d_3_, the recoveries for the polar fraction of BD (BD polar), MeOH/EtOH, and MeOH/EtOH/H_2_O after three extraction procedures were 77 ± 2 %, 59 ± 5 % and 79 ± 2 %, respectively, for a sextuplicate experiment. A much lower recovery was obtained for MeOH/EtOH when compared to the two other extraction methods. MeOH/EtOH failed to maintain a compact protein pellet during the extraction process; therefore, an extra centrifugation was required for MeOH/EtOH extraction to remove particulates in the sample extract. Moreover, based on the selected 24 endogenous metabolites in *S. meliloti*, the extra centrifugation step in MeOH/EtOH could also have caused lower extraction efficiencies when compared to MeOH/EtOH/H_2_O solvent (see Electronic Supplementary Material Fig. S4). Minimal sample handling and short-time extraction procedures are critical for large-scale metabolomic studies in order to achieve greater reproducibility and sensitivity and to prevent metabolite modification and degradation with time [14, 30]. In terms of ease of performance, the MeOH/EtOH/H_2_O extraction procedure outperformed MeOH/EtOH and the two-staged extraction procedure of BD for comprehensive polar and nonpolar metabolite analyses. Hence, the MeOH/EtOH/H_2_O was further optimized to achieve better extraction efficiencies and robustness.

**Fig. 1:**
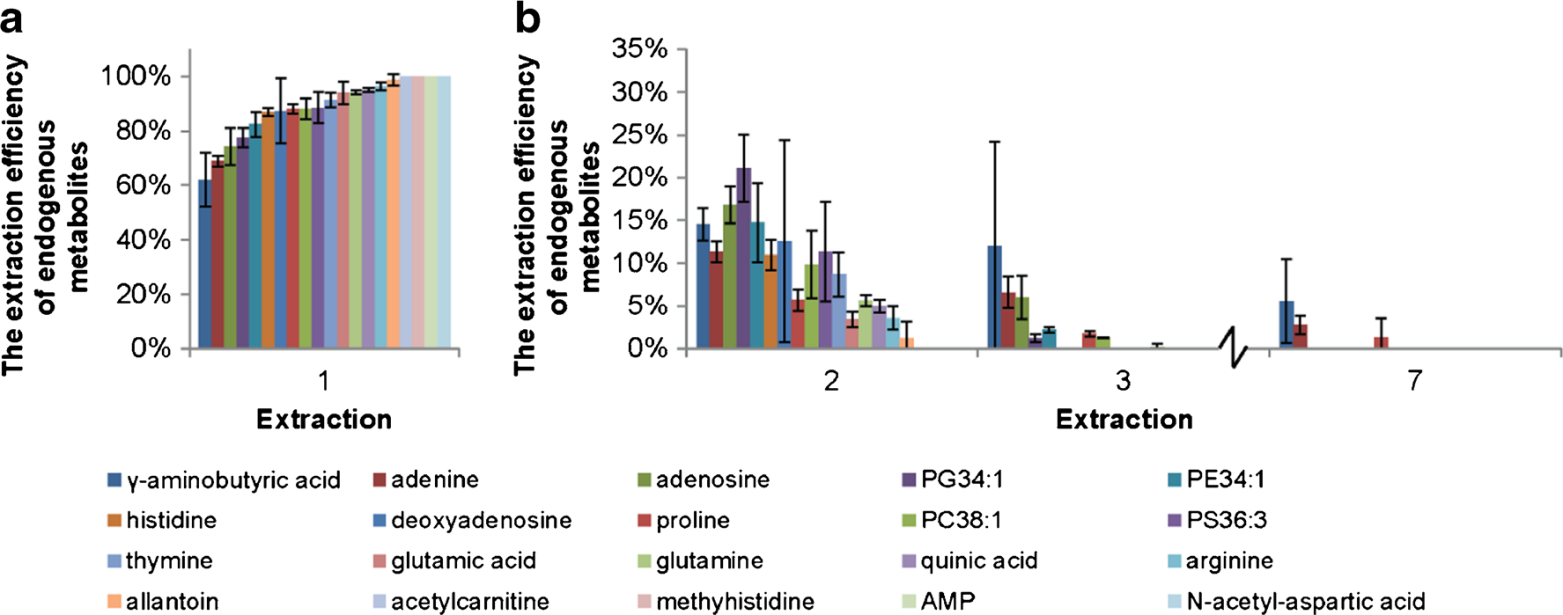
The extraction efficiencies of endogenous metabolites in 2 × 10^9^ *S. meliloti* cells. **a** The extraction efficiencies in the first extraction and **b** the extraction efficiencies were further explored with the second to the seventh extractions (only *second*, *third* and *seventh* extractions are shown). The extraction efficiencies at each extraction step were calculated by first normalizing the peak area of each metabolite with internal standards and then dividing the relative abundance of each individual metabolite at each step with its sum in all seven extractions. The *error bars* corresponded to two standard deviations. *AMP*, adenosine monophosphate

The optimal numbers of extraction processes required in order to reach a minimal of 95 % extraction efficiency of the selected endogenous metabolites was determined by extracting a 100-μL *S. meliloti* cell culture using MeOH/EtOH/H_2_O seven times. Each extraction was performed in sextuplicate. The percentages of recoveries of 20 endogenous metabolites from *S. meliloti* at each extraction step were calculated by dividing the relative abundance of each individual metabolite at each step with its sum in all seven extractions (Fig. 1). Most metabolites showed greater than 80 % recovery upon the first extraction. Among them, *N*-acetyl-aspartic acid, adenosine monophosphate (AMP), methylhistidine and acetylcarnitine were entirely (100 %) recovered in the first extraction. The second extraction was able to recover the remaining 10–15 % for most of the metabolites. Metabolites such as γ-aminobutyric acid, adenine, adenosine and proline were persistent and were still detected after the seventh extraction. Therefore, a minimum of two MeOH/EtOH/H_2_O extraction steps were required to ensure at least 95 % extraction efficiency for the major endogenous metabolites. We recommend extracting cells three times to ensure great extraction efficiency and reproducibility.

The dissolution solvent has a significant impact on peak shapes in HILIC chromatography [31]. The samples were concentrated in order to improve the detection limit. Sample volume was reduced to 50 μL by drying with a gentle stream of nitrogen, and the remaining solvent was primarily composed of water as it was the least volatile solvent in the MeOH/EtOH/H_2_O extracts. Water is not an appropriate dissolution solvent for HILIC gradients with a high percentage of ACN because it causes peak broadening and, consequently, reduces sensitivity [31]. Therefore, samples were dried completely to remove all residual water and reconstituted in a solvent mix low in water to also minimize irreproducibility due to inconsistent sample volumes. We have adopted the use of 60 %*v*/*v* ACN/H_2_O to ensure adequate peak shape and sensitivity while still allowing full dissolution of the highly polar metabolites.

### HILIC/MS for simultaneous detection of both polar and lipid metabolites

HILIC is typically used to separate polar compounds via hydrophilic partitioning mechanism. In 2010, HILIC was reported to be able to retain lipids, especially phospholipids, according to the polarity of the lipid heads [32, 33]. Therefore, since HILIC can simultaneously separate polar and lipid metabolites, it was selected as the chromatographic method for high-throughput comprehensive metabolomic analyses. RPLC is often used to retain and separate nonpolar analytes [34]. Separation of polar compounds can also be achieved with RPLC with an ion-pairing agent in the mobile phase [35]; however, the ion-pairing reagents often lead to contamination in the MS instrument [36] and, therefore, were not preferred.

Unlike RPLC, small changes in pH and buffer ionic strength can often cause large retention deviations in HILIC [37]. To improve reproducibility and minimize retention deviation for better retention time alignment using XCMS, consistent preparation of the mobile phase was critical. For large-scale comprehensive metabolomic analyses, all samples should be run using the same batch of mobile phases. The IS and RS spiked in each biological samples should also be used to correct retention time drift of metabolites when assigning metabolite identification based on the retention time of authentic standards. HILIC separations are less tolerant of fast gradients and require a longer equilibrium time compared to RPLC. Though the starting gradient at 98 % ACN was able to retain a greater amount of metabolite features, it required much longer equilibration time (more than 10 min) than starting at 95 % ACN (8–10 min). Running blanks and pooled samples at the beginning of the HILIC sequence is critical in order to condition the column to minimize variation in peak shape, retention time and ionization response.

**Fig. 2:**
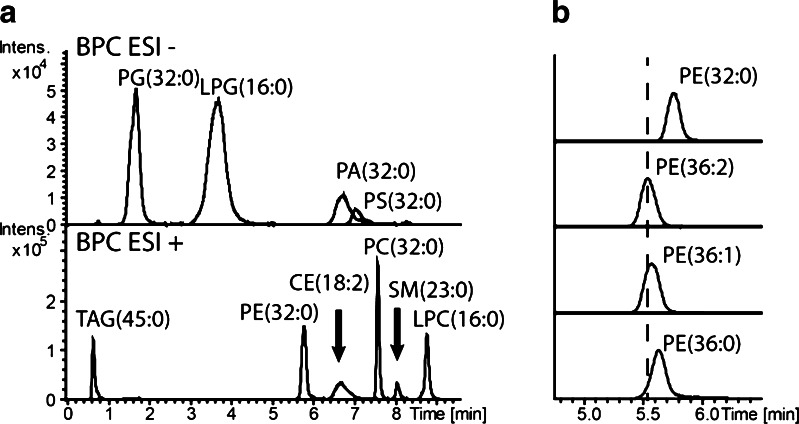
**a** Base peak chromatogram of the HILIC separation on ten lipid classes including *PG*s, lyso-PG, phosphatidic acids (*PA*s), *PS*s, triacylglycerols (*TAG*), PEs, ceramides (*CE*s), *PC*s, sphingomyelins (*SM*s) and lyso-PCs. **b** Base peak chromatogram of [M+H]^+^ ions of PEs

**Fig. 3:**
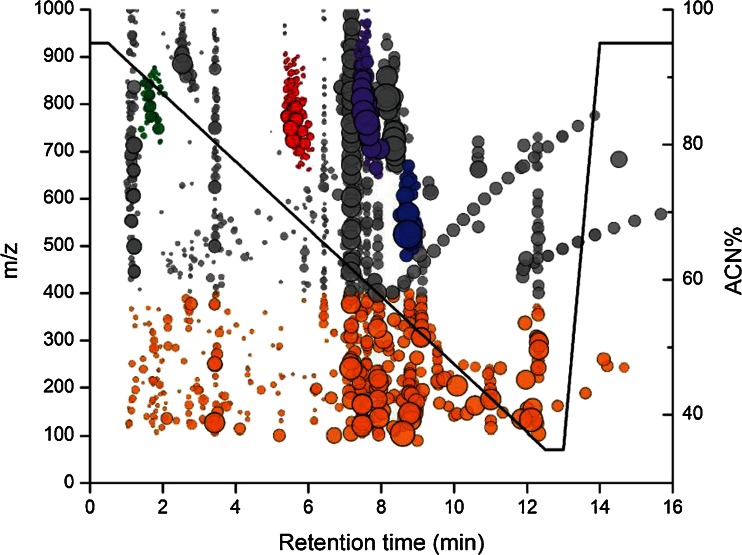
The 2,125 endogenous metabolite features detected in 3 × 10^5^ murine macrophage extracts after data reduction. The radius of the data markers (*filled circle*) reflected the relative abundances of the metabolite features over a dynamic range of 5 × 10^4^. Small metabolites (90–400 *m*/*z*) in *orange*, PGs in *green*, PEs in *red*, PCs in *purple* and lyso-PCs in *blue* were highlighted. The HILIC gradient was labelled with a *black line* with reference to percentage acetonitrile

HILIC is able to retain phospholipids or other polar lipids via an adsorption mechanism [32]. Silica HILIC was chosen specifically for optimized phospholipid separation at low buffer strength instead of other commonly used zwitterionic or diol HILIC [32, 38]. The low buffer ionic strength of 10 mM ammonium acetate in mobile phase B allowed secondary interactions between the HILIC stationary phase and the polar lipid head groups via hydrogen bonding and electrostatic interaction. Therefore, our optimized HILIC gradient was able to separate phospholipids by classes based on their polar head group. Though each lipid class eluted within a very narrow time window (often within 1 min), there were still separations within each lipid class based on hydrophobicity (carbon chain length) and unsaturation (number of C=C bonds) (Fig. 2). The lipid class separation achieved with HILIC in combination with the sub-5-ppm mass accuracy attained with internal calibration was able to accurately identify lipid metabolites without running copious authentic standards. Isomers between phosphatidylcholine (PC) and phosphatidylethanolamine (PE) could be accurately identified because PCs and PEs were chromatographically separated. Figure 3 summarizes 2,125 metabolite features (after data reduction) detected in the intracellular extracts of murine macrophage. Different classes of phospholipids including phosphatidylglycerol, PCs, PEs and lyso-PCs were detected along with small polar metabolites such as nucleosides, amino acids and organic acids. The ability of HILIC to separate both polar and lipid compounds combined with our extraction methodology allowed simultaneous analyses of both polar and lipid metabolites for enhanced sample throughput.

Mass accuracy can be significantly improved by the usage of sodium formate as an internal calibrant which was formed by the endogenous sodium ions in the cell and the formic acid in mobile phase B. The presence of sodium formate adducts with retention times at 7.2 min was used for internal mass calibration in both ESI+ and ESI− modes which dramatically improved mass accuracies for all three cell types grown in different biological media (see Electronic Supplementary Material Table S2). The confidence of metabolite identification was improved significantly with the sub-5-ppm mass accuracy attained after internal calibration with sodium formate.

The endogenous sodium formate also caused minor ion suppression regardless of the biological matrix of interest (see Electronic Supplementary Material Fig. S5). The IS, phenylalanine-d_8_, eluted in the ion suppression region and was used to normalize peak areas of metabolite features eluting in the region to correct for varying degrees of ion suppression in different samples.

The current method was applied to the comprehensive metabolomic analyses of *S. meliloti*, *S. intermedius* and murine macrophages. Over a continuous 7-day injection series of *S. meliloti* extracts, the retention time deviation was less than 7 s in a 24-min LC run with approximately 260,000 metabolite features in 137 samples (1,900 features per sample over 137 samples in ESI+) analysed by XCMS using the centWave method (see Electronic Supplementary Material Fig. S6). The peak area deviations of IS were all below 10 %. The QC and sample data were analysed with PCA with QC samples clustered tightly in the centre of the score plot, indicating that instrumental variability was minimal. The optimized HILIC-TOF-MS method was highly robust and reproducible.

### Metabolome coverage from the MeOH/EtOH/H_2_O extraction compared to the two fractions of the Bligh and Dyer method

Untargeted comprehensive large-scale metabolomics demands that the experimental method have high sample throughput, high robustness to sustain long LC sequence and excellent metabolome coverage. We propose using a MeOH/EtOH/H_2_O extraction in combination with HILIC-TOF-MS to encompass both polar and nonpolar metabolites in a single analysis. Compared to the conventionally used BD method in which polar and nonpolar fractions are analysed separately, the proposed method doubles the throughput and minimizes the sample handling time with comparable reproducibility. The metabolome coverage of the proposed method was compared to both of the polar and nonpolar fractions obtained using BD methods.

Traditionally, the BD polar fraction was run using HILIC, and the BD nonpolar fraction was run using RPLC [18, 19, 39]. However, in order to directly compare the extraction efficiency of nonpolar metabolites, the BD nonpolar fraction was also run using the same optimized HILIC method as used for the MeOH/EtOH/H_2_O extracted samples and BD polar extracts. Evaluating all three extract samples using the same LC method has also allowed us to compare metabolite features that were found in common between all three extracts. However, more features were expected when analysing the BD nonpolar fraction with RPLC in comparison to HILIC. Triacylglycerols, diacylglycerols and fatty acids, which were commonly analysed with RPLC, cannot be retained using HILIC, and were eluting in the unquantifiable dead volume with retention time below *k*_app_′ 0.7. Gram-negative bacteria *S. meliloti* was used to compare the metabolome coverage and extraction efficiency of MeOH/EtOH/H_2_O to BD polar and BD nonpolar extracts.

The XCMS centWave algorithm in combination with CAMERA has deconvoluted a total of 3,378 metabolite features. All those features were present in at least one of the MeOH/EtOH/H_2_O, BD polar and BD nonpolar extracts. There were more features detected in the ESI- mode (1,900) compared to ESI+ mode (1,478). Metabolite features from solvent contamination, instrumentation noise and spiked IS and RS that were shared in the extracted samples and the standard mixture containing IS and RS were removed (unpaired heteroscedastic *t* test, *p* > 0.05 between all extracts and standard mixtures). Any features that were eluted in the dead volume with *k*_app_′ < 0.7 were removed because they were unquantifiable due to severe ion suppression. The isotopic ions annotated by CAMERA were also removed along with ions associated with sodium formate clusters. After data reduction, a final list of 1,059 metabolite features was attained. The data reduction process was important to reduce the quantity of redundant data and false positives during statistical analyses.

**Fig. 4:**
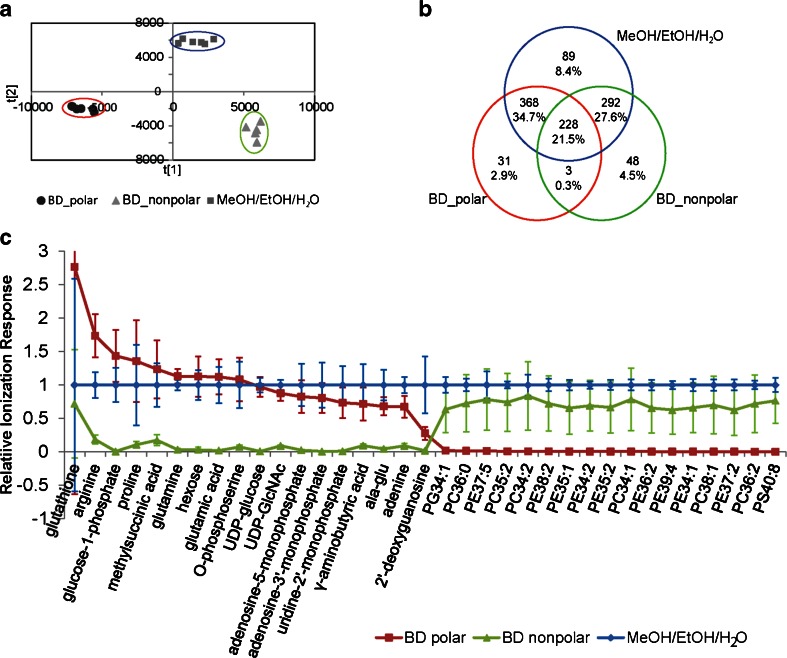
The metabolome coverage of *S. meliloti* of the 1,059 endogenous metabolite features found in at least one set of BD polar, BD nonpolar or MeOH/EtOH/H_2_O extracted samples. Extracts were performed in sextuplicate and analysed independently by HILIC-TOF-MS in both ESI+ and ESI− modes. The ionization responses of the metabolite features were normalized using internal standards. **a** OPLS-DA score plot comparing the endogenous metabolome coverage of *S. meliloti* extracts attained from three different extraction methods with *R* ^2^ *X*(cum) = 0.926, *R* ^2^ *Y*(cum) = 0.986 and *Q* ^2^(cum) = 0.936. **b** The quantity of metabolite features that were uniquely identified and shared between BD polar, BD nonpolar and MeOH/EtOH/H_2_O extracted samples was listed in the Venn diagram with their estimated percentage share of the total detectable metabolome. **c** The normalized ionization responses of identified metabolites with varying polarities. The ionization responses from MeOH/EtOH/H_2_O extracts were set to one as references. Polar metabolites were mostly extracted in BD polar, and lipids were seen exclusively in BD nonpolar; however, all metabolites were detected in MeOH/EtOH/H_2_O. *Error bars* corresponded to two standard deviations. *UDP*, uridine diphosphate; *GlcNac*, *N*-acetylglucosamine

Multivariate analysis using OPLS-DA revealed that all three types of extracts had unique metabolome profiles (Fig. 4a). The model was robust with *Q* ^2^(cum) = 0.936 and describes nearly all variables with *R* ^2^ *X*(cum) = 0.926 and *R* ^2^ *Y*(cum) = 0.986. All extracts from the MeOH/EtOH/H_2_O extraction were clustered in between the BD polar and BD nonpolar extracts, indicating shared metabolome profiles between MeOH/EtOH/H_2_O extracts and BD polar extracts as well as nonpolar extracts. Since MeOH/EtOH/H_2_O extracts were not centered in the OPLS-DA score plot, these extracts contained some unique metabolite features that were absent in the BD polar and BD nonpolar extracts.

Among the 1,059 detectable endogenous metabolite features of *S. meliloti*, 59.4, 53.9 and 92.2 % were detected in BD polar, BD nonpolar and MeOH/EtOH/H_2_O extracts, respectively (Fig. 4b). Of the 7.8 % of the detectable metabolome not covered by the MeOH/EtOH/H_2_O method, 2.9 % were only detected in BD polar, 4.5 % were only detected in BD nonpolar and 0.3 % were detected in both BD polar and nonpolar fractions. There were 34.7 % of the features shared between MeOH/EtOH/H_2_O and BD polar that were undetected in BD nonpolar; among them, polar metabolites such as amino acids, organic acids, sugar phosphates, nucleotides and nucleosides were detected. There were 27.6 % of the features shared between MeOH/EtOH/H_2_O and BD nonpolar that were not detected in BD polar; lipids such as phosphatidylglycerols (PGs), PEs, PCs and phosphatidylserines (PS) were among those that were identified. There were 8.4 % of the metabolite features that could only be detected in MeOH/EtOH/H_2_O extracts, and they ranged in polarity and *m*/*z* values. A few of the identified metabolites and their relative responses are shown in Fig. 4c, and the ionization responses of all metabolites in MeOH/EtOH/H_2_O were normalized to one as a reference. The MeOH/EtOH/H_2_O method had equivalent recoveries for vast majority of those endogenous polar and lipid metabolites as compared to the separately analysed BD polar or BD nonpolar extracts.

**Fig. 5:**
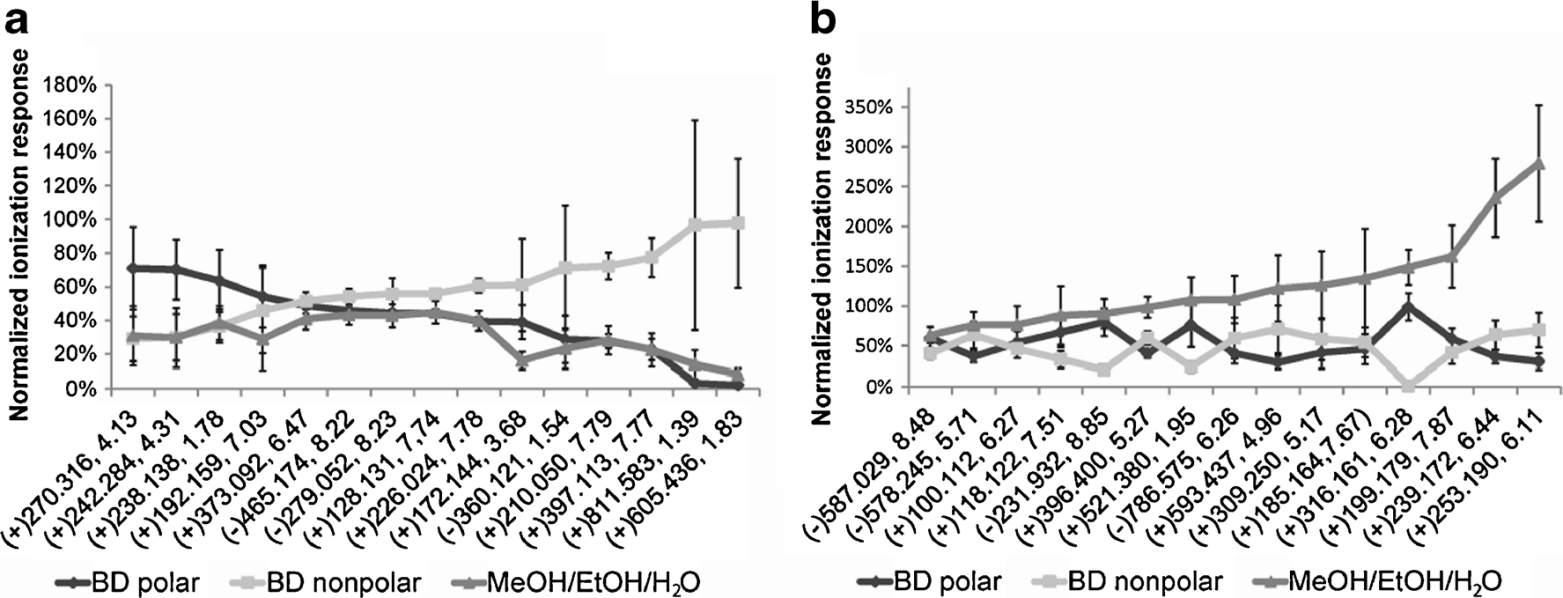
Among 228 shared metabolite features in BD polar, BD nonpolar and MeOH/EtOH/H_2_O extracts, **a** 14.6 % features had lower extraction efficiencies in MeOH/EtOH/H_2_O extracts than at least one of the BD extracts, and **b** 15.8 % features had increased extraction efficiencies in MeOH/EtOH/H_2_O extracts than all BD extracts. Each *error bar* corresponds to two standard deviations calculated from six independent extractions. The ionization responses were normalized to the IS and also the total summed ionization response for both BD polar and BD nonpolar. Each metabolite feature is represented by the ESI mode used for its detection (in *brackets*), its *m*/*z* value and retention time

There were also 21.5 % of the features shared between MeOH/EtOH/H_2_O, BD polar and BD nonpolar extracts. Among those shared features, 59.6 % of the shared metabolite features were equally extracted using MeOH/EtOH/H_2_O in comparison to the most pronounced BD fractions based on unpaired heteroscedastic Student’s *t* test of *p* < 0.05 (Table 1). In Fig. 5, the ionization responses of some selected shared metabolite features were normalized against IS and the sum of ionization response of BD polar and BD nonpolar which was normalized to 1. The normalization was done under the assumption that BD polar and BD nonpolar in combination have a net 100 % recovery of all metabolites. MeOH/EtOH/H_2_O had lower extraction efficiency in 14.6 % of the shared features than at least one of the BD fractions (Fig. 5a). However, 15.8 % of the shared features had a greater recovery in MeOH/EtOH/H_2_O than both of the BD fractions (Fig. 5b). Many of those metabolite features were partially recovered in both BD polar and BD nonpolar, but were more efficiently recovered using MeOH/EtOH/H_2_O. If a conventional comprehensive metabolomic method was used with each of the BD polar and nonpolar fractions run on either HILIC or RP, then features that were partially extracted and present in both fractions would be considered as different metabolites and would be quantified separately and result in bias during multivariate analyses. Moreover, recovering these metabolites in full using MeOH/EtOH/H_2_O results in higher injected concentrations and facilitates their detection. Therefore, the 8.4 % of the metabolite features detected exclusively in the MeOH/EtOH/H_2_O method would likely be low abundant metabolites in the cells which were under a detection limit when partially recovered in either of the BD fractions, but detectable when more efficiently recovered in the MeOH/EtOH/H_2_O extraction.

**Table 1:** The shared 228 metabolite features in BD polar, BD nonpolar and MeOH/EtOH/H_2_O extracts were compared between different extraction methods using unpaired heteroscedastic Student’s *t* test with *p* < 0.05. Among the shared features, 15.8 % showed greater extraction efficiency (↑), 14.6 % showed lower extraction efficiency (↓) and 59.6 % showed no difference (≈) between MeOH/EtOH/H_2_O and BD fractions

		Features detected	Percentage features of 228 shared	
Extraction efficiency of MeOH/EtOH/H_2_O compared to BD	↑	36	15.8	MeOH/EtOH/H_2_O > BD polar, BD nonpolar
	*↓*	*5*	*2.2*	*BD polar > MeOH/EtOH/H*_*2*_ *O > BD nonpolar*
		*6*	*2.6*	*BD polar < MeOH/EtOH/H*_*2*_ *O < BD nonpolar*
		*2*	*0.9*	*MeOH/EtOH/H*_*2*_ *O < BD polar, BD nonpolar*
		*7*	*3.1*	*MeOH/EtOH/H*_*2*_ *O = BD nonpolar < BD polar*
		*36*	*15.8*	*MeOH/EtOH/H*_*2*_ *O = BD polar < BD nonpolar*
	≈	29	12.7	MeOH/EtOH/H_2_O = BD polar = BD nonpolar
		39	17.1	MeOH/EtOH/H_2_O = BD nonpolar > BD polar
		68	29.8	MeOH/EtOH/H_2_O = BD polar > BD nonpolar

“>”, greater extraction efficiency; “<”, less extraction efficiency; “=”, equal extraction efficiency. Metabolite features seen with greater ionization responses in either BD polar and BD nonpolar fractions in comparison to those from MeOH/EtOH/H_2_O extracts were considered to have lower extraction efficiencies in MeOH/EtOH/H_2_O and vice versa

Based on all these results, MeOH/EtOH/H_2_O in combination with HILIC-TOF-MS provides a very robust, high-throughput and comprehensive approach for cellular metabolomic analyses. The metabolomic coverage of MeOH/EtOH/H_2_O was comparable to the combined coverage of BD polar and BD nonpolar yet was twice as efficient in terms of data acquisition speed. The method was unbiased towards neither of the polar or nonpolar metabolites.

## Conclusion

The complex biological matrices and different culturing techniques required for the growth of many cellular organisms present a great challenge for comprehensive analyses. Large-scale comprehensive metabolomic analyses involving hundreds of samples often lead to time-consuming, labour-intensive sample preparation, extraction and data acquisition. The proposed comprehensive metabolomic protocol using MeOH/EtOH/H_2_O extraction paired with HILIC-TOF-MS analysis was able to expand metabolome coverage to both polar and lipid metabolites with high reproducibility and robustness with twofold faster data acquisition throughput than the conventional Bligh and Dyer method coupled to RP and HILIC-MS. This scalable extraction method was applicable to Gram-positive and Gram-negative bacteria with rigid cell walls as well as mammalian cells; it was applicable to both suspension and adherent cell cultures that were grown in either rich or minimal media and had minimal ion suppression despite the complex biological matrix. This comprehensive metabolomic method was developed as a qualitative initial screening tool for finding biomarker or pathway differences between treatments for a hypothesis-generating study. However, extension to quantitative targeted metabolomic analyses should be followed for high-impact study.

## Electronic supplementary material

Below is the link to the electronic supplementary material.

ESM 1 (PDF 7973 kb)

## Abbreviations

ACN: Acetonitrile
AMP: Adenosine monophosphate
BD: Bligh and Dyer
BD nonpolar: Nonpolar fraction of BD
BD polar: Polar fraction of BD
CE: Ceramides
ESI: Electrospray ionization
ESI+: Positive electrospray ionization mode
ESI−: Negative electrospray ionization mode
EtOH: Ethanol
GlcNac: *N*-Acetylglucosamine
Gly-Phe: Glycine-phenylalanine
HILIC: Hydrophilic interaction liquid chromatography
IS: Internal standards
LC: Liquid chromatography
MeOH: Methanol
MS: Mass spectrometry
OPLS-DA: Orthogonal partial least-squares discriminative analysis
PA: Phosphatidic acid
PBS: Phosphate-buffered saline
PC: Phosphatidylcholines
PCA: Principal component analysis
PE: Phosphatidylethanolamines
PG: Phosphatidyl glycerol
Phe-Phe: Diphenylalanine
PS: Phosphatidylserines
QC: Quality control
RP: Reversed-phase
RS: Recovery standards
SM: Sphingomyelin
Snthr: Signal-to-noise threshold
TAG: Triacylglycerol
TOF: Time-of-flight
UDP: Uridine diphosphate
