# Supplementary material for: Comprehensive and simultaneous coverage of lipid and polar metabolites for endogenous cellular metabolomics using HILIC-TOF-MS
Source: Anal Bioanal Chem. 2014 Apr 10;406(15):3723–33. doi: 10.1007/s00216-014-7797-5 (PMC4026627; doi:10.1007/s00216-014-7797-5)
Supplement: Supplementary file 1 — (PDF 7973 kb) [file 216_2014_7797_MOESM1_ESM.pdf]

Analytical and Bioanalytical Chemistry

Electronic Supplementary Material

Comprehensive and simultaneous coverage of lipid and polar metabolites for endogenous cellular metabolomics using HILIC-TOF-MS

Fan Fei, Dawn M. E. Bowdish, Brian E. McCarry

1. Cell culture conditions

1.1. Cell Culture for *Sinorhizobium meliloti*

S. meliloti RmP100 (wild type strain) were streaked onto LB agar plates and incubated at 30ºC for four days. A single colony from the agar plate (less than 2 weeks old) was inoculated into 5 mL LB_{mc} rich medium at 30ºC for 24 hours. The culture was centrifuged, then washed with 1 mL of sterile saline solution (0.85% NaCl), and re-suspended in M9 culture. The re-suspended *S. meliloti* culture was inoculated to M9 at initial OD_{600} of 0.05 and incubated at 30ºC for 24 hours till reaching mid-log phase (OD_{600} = 1.0).

The LB broth (10 g/L DifcoBactoTryptone, 5 g/L Difco Yeast Extract, and 5 g/L NaCl) was adjusted with 1M NaOH to pH 7.0, and autoclaved. The LB_{mc} broth was prepared by adding 250 µL of sterile 1 M $MgSO_4$ and 500 µL of sterile 0.5 M $CaCl_2$ to 100 mL of sterilized LB broth to reach a the final concentration of 2.5 mM of both $MgSO_4$ and $CaCl_2$.

The M9 medium was prepared by adding 100 mL 5x Difco M9-salts, 0.5 mL $MgSO_4$ (1 M), 0.25 mL $CaCl_2$ (0.5 M), 5 µL $CoCl_2$ (1 mg/mL), 50 µL biotin (1 mg/mL), 5 mL glucose (1.5 M) and 394.2 mL autoclaved distilled water to reach a final volume of 500 mL. The 5x Difco M9-salts were composed of 33.9 g/L $NaHPO_4$, 15 g/L NaH_2PO_4, 2.5 g/L NaCl, and 5 g/L NH_4Cl. All solutions used were autoclaved separately prior to use. The glucose solution was sterilized by filtration through a 0.45 µmSupor membrane filter (Acrodisc). The final M9 medium was composed of 20% v/v 5x Difco M9-salts, 1 mM $MgSO_4$, 0.25 mM $CaCl_2$, 0.01 µg/mL $CoCl_2$, 1 µg/mL biotin, and 15 mM glucose.

1.2. Cell Culture for *Streptococcus intermedius*

S. intermedius B196 was streaked on Todd Hewitt agar supplemented with yeast extract and incubated for 3 days at 37ºC at 5% CO_2. A single Colony was used to inoculate Todd Hewitt broth supplemented with yeast extract (THY). Seven replicates were incubated at 37ºC. Overnight cultures were diluted tenfold in THY and grown to 0.8 OD_{600}.

S1.3 Cell Culture for Murine Macrophages

Bone marrow progenitors were isolated from the leg bones of young (6-8 weeks) C57BL/6 female mice. The progenitor cells were cultured and differentiated for 7 days at 37ºC in 150 mm Petri dishes (Fisherbrand) in 25 mL Roswell Park Memorial Institute 1640 medium (RPMI-1640) supplemented with 1% penicillin-streptomycin (P/S), 1% L-glutamine, 10% fetal bovine serum (FBS), and 15% L929-cell conditioned medium (LCM). The cell culture was changed every 2-3 days. At day 8, 3×10^5 fully differentiated macrophages wereextracted for metabolomic analyses.

2. Extraction of extracellular medium

Directly from the culture plate, 20 µL of the extracellular medium was extracted with 80 µL of MeOH/EtOH (1:1) containing RS. The solution mixtures were mixed by vortex for 2 min and centrifuged at 9500x g for 3 min. The clear supernatants were collected and diluted 2-fold in 60%v/v ACN/H_2O for MS analyses. The extracellular extracts were analyzed with the same LC-MS method as the endogenous cellular extracts.

3. Cell Washing Removes Interferences from Extracellular Medium

The sample preparation protocol was developed to quench, harvest and extract suspension and adherent cell cultures. Most prokaryotic and eukaryotic cell lines require rich growth medium to facilitate growth. However, after aspirating the cellular medium from the cell pellet, the extracellular medium that still remained at the cell surfaces can cause significant matrix effects. This extracellular fluid contained nutrients from the growth medium as well as the exogenous metabolites released from the cell. Those metabolites, if included in the endogenous cellular extracts, can suppress MS signals and also cause bias to the true endogenous metabolite concentrations [1]. In Fig. S1a, the total ion chromatogram (TIC) of the extracellular medium of murine macrophages cultured in RPMI-1460 medium qualitatively resembled the TIC of the respective endogenous cellular extracts when the cells were not washed with PBS or saline. The largely abundant nutrients and exogenous metabolites in the growth medium masked the lesser abundant endogenous cellular metabolites. With one PBS or saline wash, the TICs of endogenous cellular extracts were completely different than those from the extracellular medium (Fig. S1b), which indicated the removal of most extracellular metabolites traces in the endogenous cellular extracts. PBS or saline was selected for cell washing in order to maintain the ionic strength balance inside and outside the cells to avoid leakage [2]. A one-time wash of the cells with PBS or saline solution was able to remove metabolite traces from extracellular medium on the cell surface.

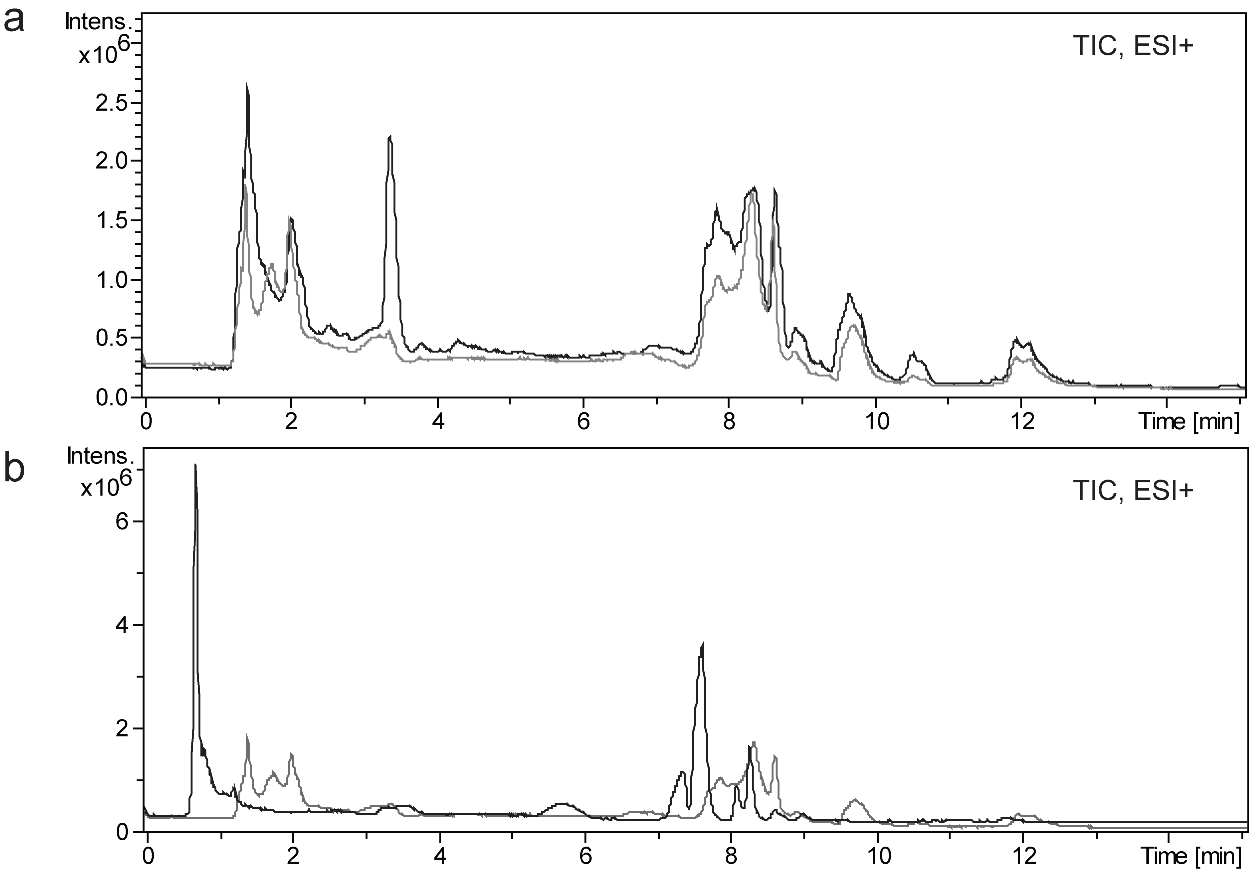

Fig. S1. The total ion chromatograms of endogenous (black) and exogenous (grey) metabolomic profiles from murine macrophages cultured in RPMI-1460. MeOH/EtOH/H_2O (2:2:1) was used to extract the endogenous metabolites. For exogenous metabolome, 20 μL of cell supernatant were extracted with 80 μL of MeOH/EtOH once and then diluted 2 fold for LC analysis. (a) The murine macrophage cells were not washed with phosphate buffered saline (PBS) prior to extraction. (b) The murine macrophage was washed once with PBS after harvest and then extracted

4. Metabolism Quenching

Ice-cold PBS or saline was used to halt cellular metabolism. Typical quenching solutions such as methanol were not used to avoid cell leakage [2]. Liquid nitrogen flash freezing was not used to avoid the impact of cold shock on cellular metabolism [3]. Incubating cells at 5ºC has shown greater reduction in cellular activity when compared to incubating in its physiological temperature [4]. Cold isotonic saline (0.9 w/v NaCl, 0.5ºC) was reported to halt ATP to ADP and AMP conversion without damaging cells [5]. In addition, the cells were extracted with cold extraction solvent immediately following PBS/saline washes to minimize further metabolic disturbances. The entire sample preparation procedure was performed on ice or in a cold room to avoid metabolite modifications and degradation.

5. Cell Detachment for Adherent Cell Culture

Harvesting cells from suspension cultures for extraction was straightforward. The cells were centrifuged, the extracellular medium was aspirated, and the cell pellet was re-suspended and washed with PBS or saline, and then extracted using an extraction solvent.

For adherent cell cultures, the cell harvest was more challenging. Typically, adherent cells were detached from their growth surface via trypsinization and then centrifuged to form a cell pellet [6]. However, trypsinization altered the integrity of the cells and their extracellular environment [7,8] which consequently led to the metabolic alternations that were not specific. Furthermore, the salts and ethylenediaminetetraacetic acid (EDTA) in trypsin buffer were not ESI-MS compatible due to its high salt concentration. To address this issue, the adherent cells were washed and quenched with cold PBS/saline, and detached by physical scraping in the presence of an extraction solvent. The organic component of the extraction solvent was able to induce cell leakage and cell death, and the cell mixture was collected and extracted. The cell detachment procedure required less than 30 seconds per sample to lift the cells compared to the much more time consuming trypsinization procedure which typically requires several minutes of incubation. Also, Bi *et al*. have shown the scraping method results in a greater metabolite recovery compared to the trypsinization method due to unrecoverable metabolite leakage experienced by the latter method [8].

6. Further Optimization of HILIC Method

The HILIC gradient was optimized using a DoE orthogonal factorial design approach to maximize metabolome coverage (Fig. S2). The DoE approach was able to simultaneously optimize many experimental parameters with a significantly reduced number of required experiments [9]. A HILIC gradient with acetonitrile (solvent A) and 10 mM ammonium acetate in water adjusted to pH3 with formic acid (solvent B) was used. The optimization was based on a generic HILIC gradient starting with an initial hold at a high percentage of A, then decreased linearly to 35% A, held isocratically at 35% A, and then followed with equilibrium back to the initial percentage of A. Three experimental factors were selected and optimized with the

following experimental ranges: initial hold time at 95% A was adjusted between 0.5 to 2.0 min, the initial percentage of A was adjusted from 80% to 98%, and the gradient from initial high percentage of A to 35% A was set to decrease with a rate from 4% to 12% of A per min. The goal of this optimization was to minimize the number of un-retained metabolite features with k_{app}' < 0.7 and also to minimize the time required for all features to elute within the gradient. The LC method was designed to ensure the elution of all features during the gradient prior to the isocratic hold at 35% A in order to minimize band broadening effects.

The hold time (X1) did not reduce the number of un-retrained features (Y1), but significantly reduced the time required to complete metabolite elution (Y2). It was clear at 98% initial ACN with a 0.5 min hold followed by a drop in gradient to 35% ACN at 4% ACN per min provided that most optimum separation, for which we observed the least amount of un-retained features (y1) and required the least amount of time (y2) to completely elute all metabolites. However, the 4% gradient slope did not take advantage the separation capacity of the entire chromatography, and the features eluted ca. 3 min prior to the end of the gradient. Moreover, 98% initial ACN required longer than 10 min equilibration time. Lastly, the optimized gradient was set to be 95% ACN with 0.5 min hold, followed by a drop in gradient to 35%ACN at a rate of 5% ACN per min. The finalized HILIC-LC gradient is shown in Fig. 2.

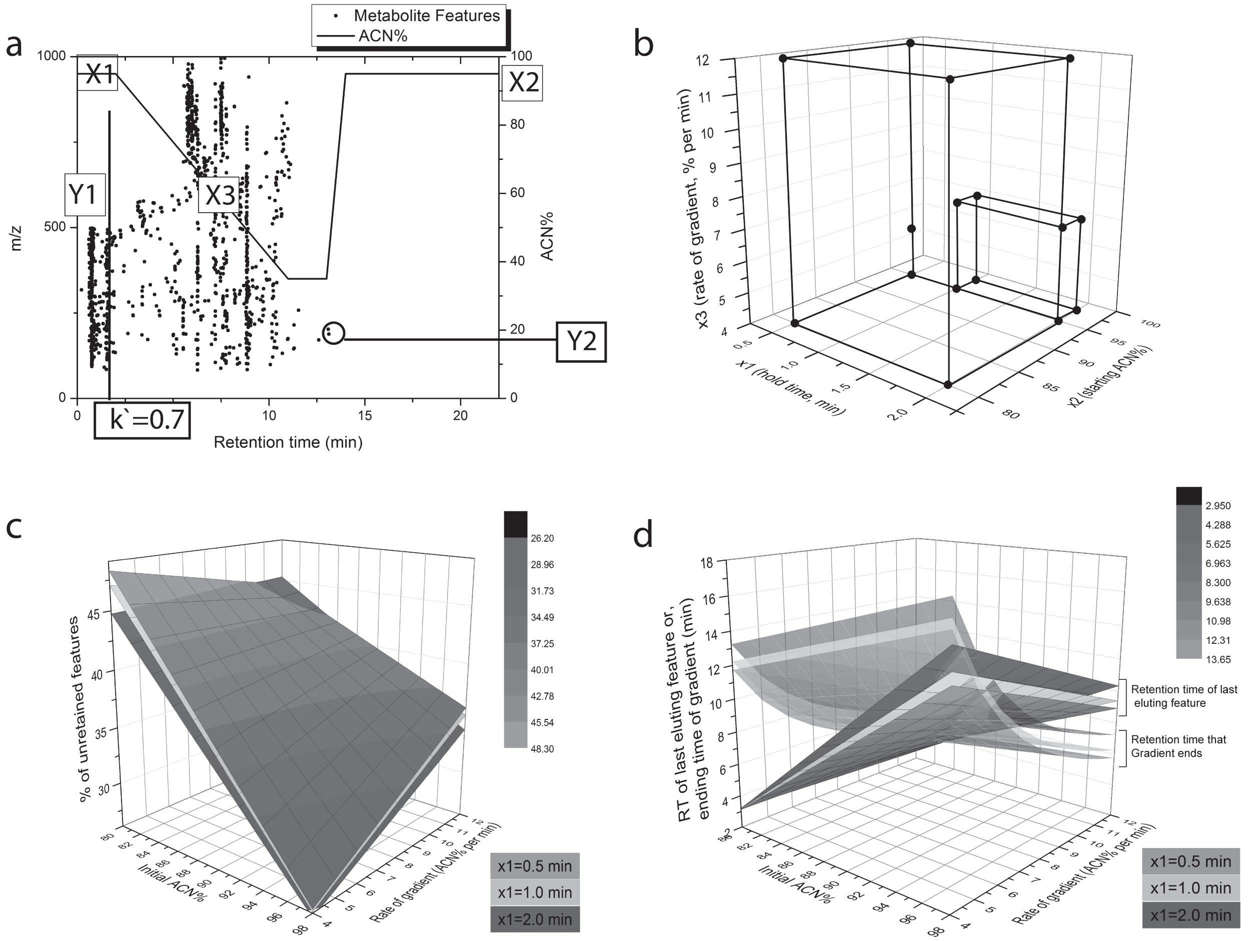

Fig. S2. 2^3 full factorial design of experiment for HILIC chromatography optimization. A HILIC gradient with acetonitrile (solvent A) and 10 mM ammonium acetate in water adjusted to pH3 with formic acid (solvent B) was used. *S. meliloti* extracts (2×10^9 cells) were separated on 50 mm × 2.1 mm Kinetex 2.6 µm HILIC column and detected using a Bruker MicrOTOF II. (A) The HILIC gradient was optimized with initial hold time (X1), starting acetonitrile percentage (X2), and the rate of change of acetonitrile percentage (X3) in order to maximize the number of metabolite features with k' < 0.7 (Y1) and minimize the time required for all features to elute during the gradient (Y2). (B) Two 2^3 factorial design was used. The axial points were repeated in triplicates, and the center point was repeated in sextuplicate. Mathematical modelled regressions based on the 2^3 full factorial designs are shown in (C) and (D) as 3D surface plots for Y1 and Y2 respectively

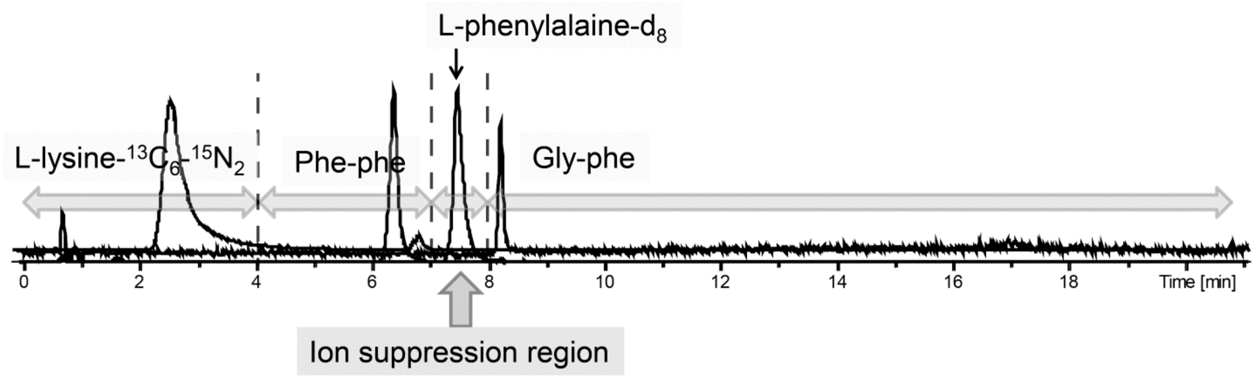

Fig. S3. The schematic for normalizing the ionization efficiency of endogenous metabolite features using L-lysine-$^{13}C_6$-$^{15}N_2$, phe-phe, L-phenylalanine-d_8 and gly-phe as internal standards. The metabolite features were normalized with internal standard that eluted closest to their retention time

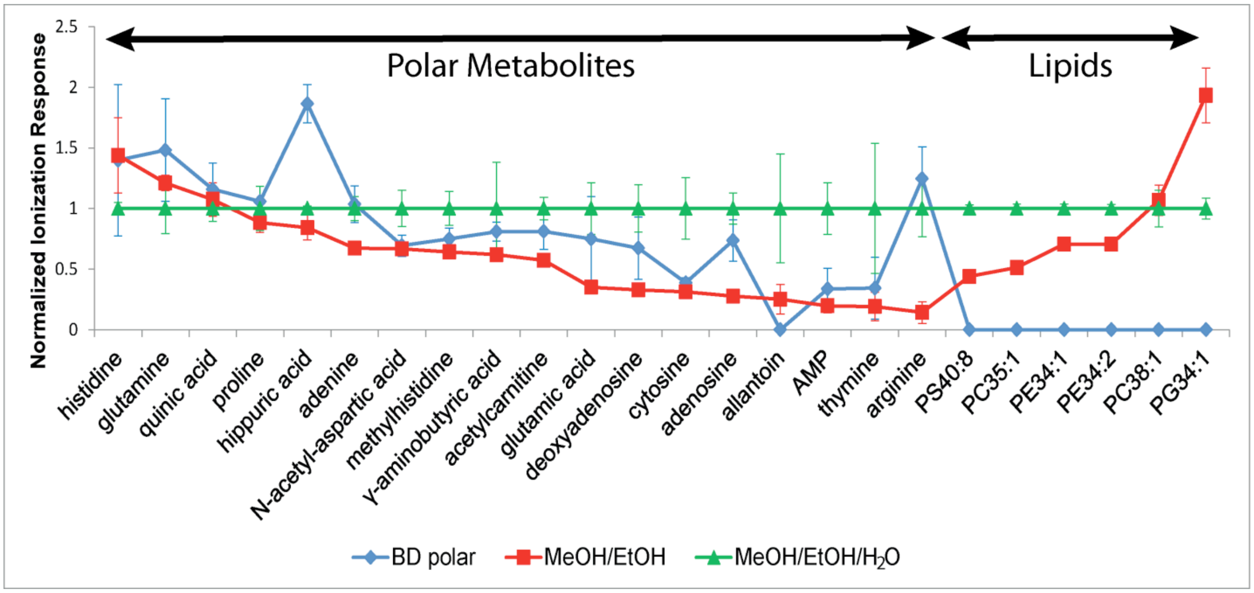

Fig. S4. The normalized ionization responses of *S. meliloti* endogenous metabolites found in BD polar, MeOH/EtOH and MeOH/EtOH/H_2O extracts. Error bars corresponded to two standard deviations based on a triplicate independent extractions normalized to the IS

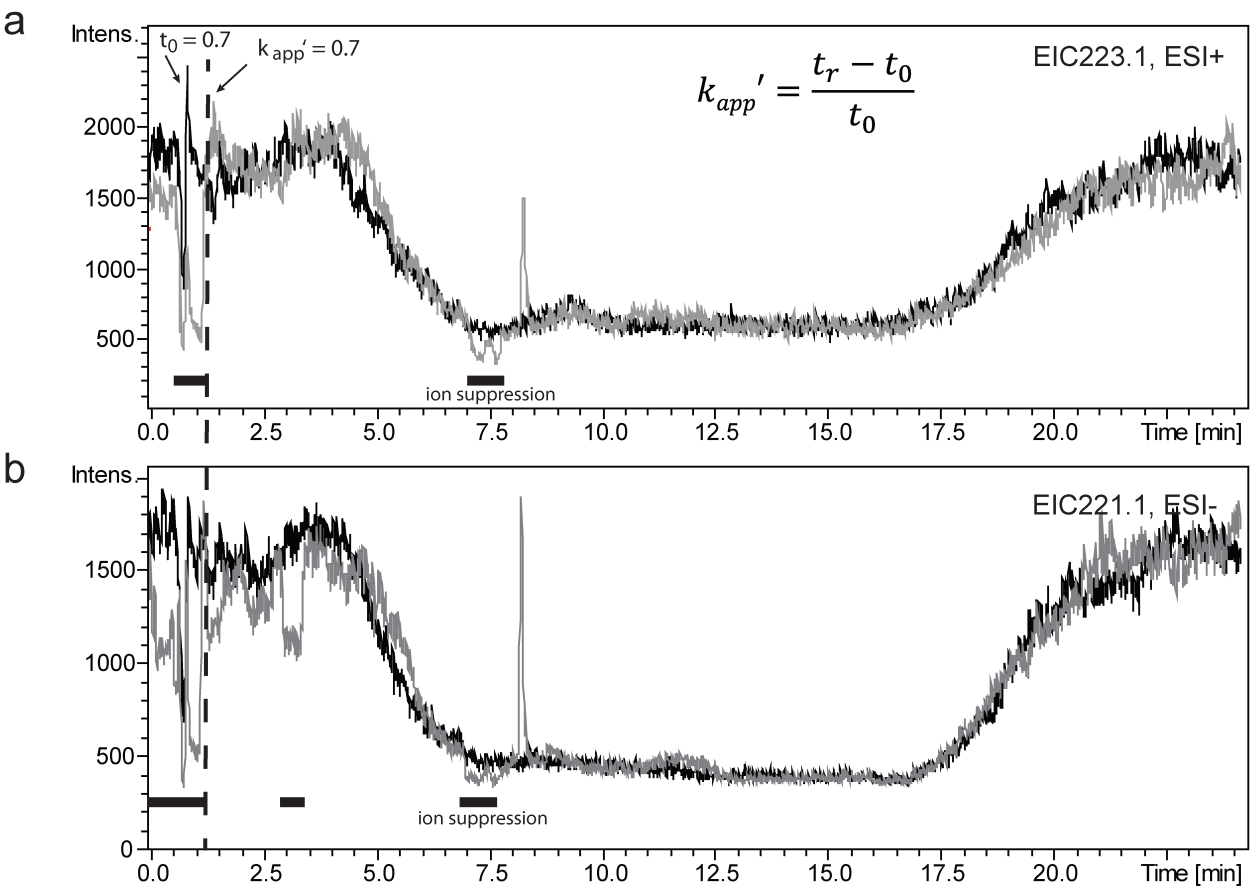

Fig. S5. The extracted ion chromatogram (EIC) of (a) $[M+H]^+$ or (b) $[M-H]^-$ ion of gly-phe added post-column following HILIC separation of endogenous murine macrophage MeOH/EtOH/H_2O extract (grey) or a 60%v/v ACN blank (black). Ion suppressions (black bold lines) were found at 0.5-1.2 min (k'_{app}: -0.3-0.7) and 6.9-8.0 min (k'_{app}: 8.9-10.4) in ESI+ mode; 0-1.2 min (k'_{app}: -0.3-0.7), 2.9-3.5 min (k'_{app}: 3.2-4.0), and 6.9-8.0 min (k'_{app}: 8.9-10.4) in ESI- mode. The gly-phe was prepared to examine whether the matrix is able to suppress ionization signals, not for quantification or quality control purposes

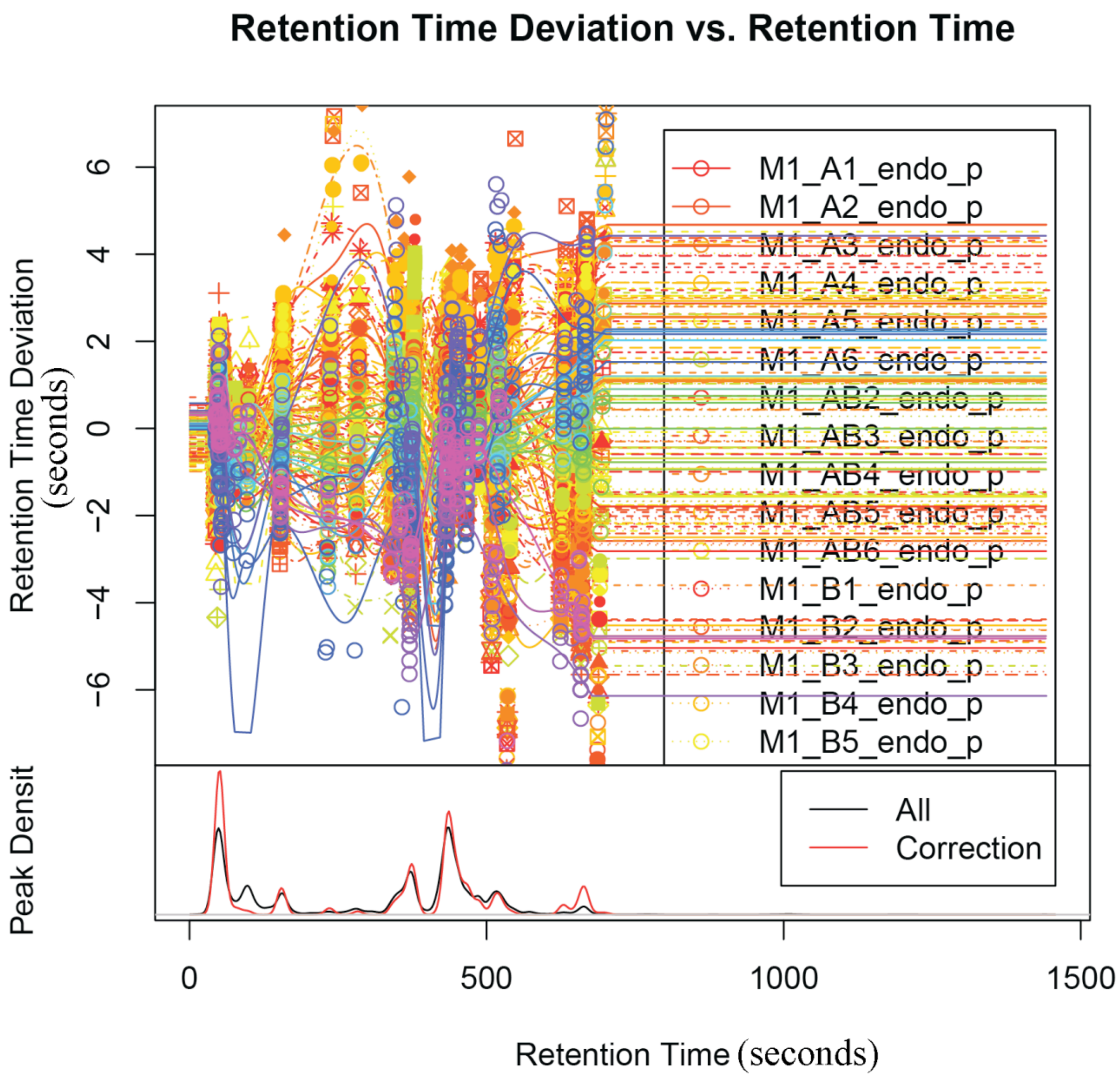

Fig. S6. The retention deviation of ca. 260,000 metabolite features detected in 137 *S. meliloti* MeOH/EtOH/H_2O extracts and pooled samples in ESI+ mode was between ±6 seconds. (Figure exported directly from XCMS)

Table S1. Metabolite features identified based on the accurate m/z values and retention time attained from authentic standards

	ESI modes	m/z	retention time (min)
2'-deoxyguanosine	neg	266.088	1.93
acetylcarnitine	pos	204.104	8.39
adenine	pos	136.076	2.05
adenosine	pos	268.127	1.75
ala-glu	neg	217.083	8.97
allantoin	pos	159.090	9.14
AMP	pos	348.068	10.04
arginine	pos	175.132	10.70
cytosine	pos	112.052	2.84
deoxyadenosine	pos	252.110	1.74
glucose-1-phosphate	pos	261.039	9.75
glutamic acid	pos	148.074	8.68
glutamine	neg	145.079	8.73
glutathione	neg	306.076	8.22
hexose	neg	179.056	1.56
histidine	pos	156.090	11.10
methylhistidine	pos	170.073	9.09
methylsuccinic acid	neg	131.031	1.57
N-acetyl-aspartic acid	neg	174.094	4.28
O-phosphoserine	pos	186.016	8.86
proline	pos	116.083	8.24
quinic acid	pos	193.119	7.21
thymine	pos	127.053	1.47
UDP-GlcNAc	neg	606.075	8.61
UDP-glucose	neg	565.048	8.47
uridine-2'-monophosphate	pos	325.044	9.38
γ-aminobutyric acid	pos	104.071	8.46
PC34:1	pos	760.581	7.54
PC34:2	pos	758.565	7.53
PC35:1	pos	774.634	6.12
PC35:2	pos	772.581	7.52
PC36:0	pos	790.612	7.50
PC36:2	pos	786.598	7.50
PC38:1	pos	800.600	7.40
PE34:1	pos	718.579	5.73
PE34:2	pos	716.557	5.69
PE35:1	pos	732.553	6.01
PE35:2	pos	730.535	5.97

PE36:2	pos	744.553	5.77
PE37:2	pos	758.567	5.95
PE37:5	pos	752.521	5.98
PE38:2	pos	770.526	6.01
PE39:4	pos	782.532	5.77
PG34:1	neg	747.547	1.67
PS40:8	neg	830.492	5.98

Table S2. The mass accuracies (ppm) of selected metabolites in different biological matrix ranging from Gram-positive *Streptococcus intermedius*, Gram-negative *Sinorhizobium meliloti*, to murine macrophages before and after internal calibration using endogenous sodium formate. The mass accuracies after the internal calibration with sodium formate were in bold

			Mass Accuracy (ppm) (before/**after** internal calibration)		
	**RT (min)**	**M+H/ M+NH4***	**murine macrophages**	***Streptococcus intermedius* (Gram-neg.)**	***Sinorhizobium meliloti* (Gram-pos.)**
adenine	2.0	136.0623	75.6/**10.5**	65.1/**10.5**	48.4/**10.1**
glutamic acid	8.8	148.0610	82.5/**16.8**	42.0/**1.6**	56.8/**2.1**
methionine-d_3	7.9	153.0777	67.1/**1.8**	56.3/**0.9**	42.9/**2.4**
phenylalanine-d_8	7.5	174.1370	65.6/**1.0**	60.3/**4.2**	44.7/**2.2**
tryptophan-d_5	7.4	210.1291	45.9/**17.8**	52.3/**5.4**	42.3/**1.5**
glycyl-phenylalanine	8.2	223.1083	64.8/**2.2**	59.4/**2.8**	46.7/**3.3**
phenylalanine-phenylanaline	6.4	313.1552	53.9/**5.3**	51.4/**2.1**	40.2/**2.9**
PC(34:2)	7.6	758.5694	44.1/**1.1**	37.6/**4.1**	31.6/**0.8**
PG(36:2)	1.8	792.5749*	51.0/**6.2**	48.7/**8.4**	38.5/**8.6**

7 References

1. Wu Y, Li L (2013) Anal Chem 85:5755–5763.

2. Bolten CJ, Kiefer P, Letisse F, Portais J-C, Wittmann C (2007) Anal Chem 79:3843–3849.

3. Wittmann C, Krömer JO, Kiefer P, Binz T, Heinzle E (2004) Anal Biochem 327:135–139.

4. Widdows J (1973) Neth J Sea Res 7:387–398.

5. Dietmair S, Timmins NE, Gray PP, Nielsen LK, Krömer JO (2010) Anal Biochem 404:155–164.

6. Lane AN, Fan TW-M (2007) Metabolomics 3:79–86.

7. Teng Q, Huang W, Collette TW, Ekman DR, Tan C (2009) Metabolomics 5:199–208.

8. Bi H, Krausz KW, Manna SK, Li F, Johnson CH, Gonzalez FJ (2013) Anal Bioanal Chem 405:5279–5289.

9. Gruendling T, Guilhaus M, Barner-Kowollik C (2009) Macromol Rapid Comm 30:589–597.
